# Electrospun Carbon Nanofibers Derived from Polyvinyl Alcohol Embedded with Bimetallic Nickle-Chromium Nanoparticles for Sodium Borohydride Dehydrogenation

**DOI:** 10.3390/polym16243541

**Published:** 2024-12-19

**Authors:** Ayman Yousef, Ibrahim M. Maafa, Ahmed Abutaleb, Saleh M. Matar, Ahmed A. Alamir, M. M. El-Halwany

**Affiliations:** 1 Department of Chemical Engineering, College of Engineering and Computer Sciences, Jazan University, Jazan 11451, Saudi Arabia; imoaafa@jazanu.edu.sa (I.M.M.); azabutaleb@jazanu.edu.sa (A.A.); sel-saidmatar@jazanu.edu.sa (S.M.M.); 2 Engineering and Technology Research Center, Jazan University, P.O. Box 114, Jazan 82817, Saudi Arabia; 3 Jazan Education Department, Jazan 45142, Saudi Arabia; 4 Department of Mathematics and Physics Engineering, Faculty of Engineering, Mansoura University, El-Mansoura 35516, Egypt; mmelhalwany@yahoo.com; 5 Faculty of Engineering, Mansoura National University, Gamasa 7731168, Egypt

**Keywords:** electrospinning, bimetallic, nickel-chromium, carbon nanofiber, hydrogen

## Abstract

Bimetallic NiCr nanoparticles decorated on carbon nanofibers (NiCr@CNFs) were synthesized through electrospinning and investigated as catalysts for hydrogen generation from the dehydrogenation of sodium borohydride (SBH). Four distinct compositions were prepared, with chromium content in the catalysts ranging from 5 to 25 weight percentage (wt%). Comprehensive characterization confirmed the successful formation of bimetallic NiCr@CNFs. Notably, among the compositions, the catalyst containing 20 wt% Cr exhibited the highest efficiency in SBH dehydrogenation. Kinetic studies revealed that hydrogen production followed a first-order reaction with respect to the catalyst quantity. Additionally, the reaction time decreased with increasing temperature. The activation energy (Ea), entropy change (ΔS), and enthalpy change (ΔH) were calculated as 34.27 kJ mol^−1^, 93.28 J mol·K^−1^, and 31.71 kJ mol^−1^, respectively. The improved catalytic performance is attributed to the synergistic interaction between Ni and Cr. This study proposes a promising strategy for the advancement of Ni-based catalysts.

## 1. Introduction

The significance of hydrogen (H_2_) as an energy carrier is growing with society’s progress toward a sustainable energy future. H_2_ has substantial potential to contribute to the decarbonization of our economy and meet our energy requirements sustainably [1,2]. The advancement of H_2_ energy sources represents a dynamic and rapidly evolving area of research, with technological advancements anticipated to reduce associated costs and expand the infrastructure required for H_2_ production, transportation, and storage [3,4]. These advancements will enhance the feasibility of H_2_ energy as a sustainable alternative for future energy needs.

Metal hydrides are considered optimal for H_2_ storage due to their ability to securely, compactly, and efficiently store H_2_ [5]. The generation of H_2_ from these materials can serve as a potential fuel or energy source. Sodium borohydride (SBH) is a promising solid crystalline hydride used for H_2_ production. With its high theoretical H_2_ capacity (10.8%) and environmentally friendly properties, SBH has been garnering significant attention in proton exchange membrane fuel cell applications for powering various electronic devices, including vehicles, smartphones, and tablets [6,7]. However, SBH exhibits instability in aqueous solutions at room temperature, leading to slow decomposition. Storing SBH in alkaline media mitigates flammability and toxicity hazards, improves stability, and results in lower reaction rates [8,9]. Therefore, it is imperative to identify suitable catalysts to enhance the rate of hydrolysis reactions. The search for an efficient catalyst capable of rapidly generating pure H_2_ from SBH has attracted increasing attention in contemporary research [10].

Noble metals have long been considered excellent choices for the dehydrogenation of SBH; however, their practical application remains significantly constrained by their high cost and limited availability, making them challenging for economical application [11,12]. Consequently, there is a growing emphasis within the scientific community on developing catalysts using more abundant metals, particularly transition metals (e.g., Co [9], Ni [8], and Cu [13,14,15]), which are not only inexpensive but also readily available and have shown promise in SBH dehydrogenation. Among the various transition metals, Ni-based catalysts are considered particularly promising due to their electronic structure and favorable stability [16]. Nonetheless, due to their higher surface energy, Ni nanoparticles tend to oxidize in air or water and are susceptible to magnetism-induced properties and agglomeration [17,18,19], which limit their catalytic activity and reduce recycling efficiency. To address these challenges, researchers have explored various strategies to stabilize Ni nanoparticles and enhance H_2_ production rates. One effective strategy involves using supported Ni nanoparticles on a variety of matrices, such as zeolites, metal oxides, polymers, and nanocarbons, to improve dispersion and prevent aggregation of nanoparticles [20,21,22]. My research group has identified carbon nanofibers (CNFs) as exceptional supports for Ni nanoparticles, offering a favorable surface for effective nanoparticle attachment [23]. Moreover, CNFs demonstrate commendable chemical and thermal stability, along with significant adsorption capacity [24,25]. The nanoporous structure of electrospun NFs facilitates the movement of reactants and products, allowing CNFs to readily adsorb SBH, thereby enhancing contact with the catalyst surface and promoting efficient H_2_ release. Another strategy involves incorporating an atomic diffusion barrier, such as Cr, Mo, or W, alongside Ni atoms [26].

Research has indicated that bimetallic nanoparticles demonstrate superior catalytic behaviors in various chemical reactions compared to their monometallic counterparts [27,28,29]. This phenomenon is thought to arise from the effects of lattice geometry, strain, and electronic charge transfer. Furthermore, this combination has the potential to produce highly active bimetallic catalysts by leveraging the interaction of electronic and lattice effects in alloy catalysts [30]. The synthesized alloy can activate the cleavage of O-H bonds in H_2_O molecules [31,32]. Additionally, the geometric properties of these bimetallic catalysts can be modified to enhance specific surface area and expand the range of active sites [30,33]. An experiment by Li H. et al. [34] demonstrated that the addition of small amounts of Cr or W to Co-B made it significantly more hydrogenated for glucose. Similarly, Patel N. et al. [35] revealed that incorporating an atomic diffusion barrier (such as Cr, Mo, or W) had a substantial impact on the catalytic performance of Co-B in the dehydrogenation of SBH. The amalgamation of these metals, even at minimal atomic concentrations, significantly enhances the surface area of the Co-B catalyst by preventing agglomeration. Additionally, they play a crucial role in facilitating the overall catalytic reaction by serving as acidic sites that promote the adsorption of reactant species on the surface. The secondary metal functions as an electron-donating ligand, thereby increasing the electron density on the active metal atoms and enhancing reaction kinetics.

Moreover, Chen L. F. et al. [36] demonstrated that adding Mo and W to Ni-B improved its activity and selectivity in breaking down p-chloronitrobenzene. In a recent study by Kangkang Yang et al. [26], it was discovered that incorporating Cr, Mo, and W into Ni nanoparticles improved their ability to dehydrogenate ammonia borane and hydrazine borane. This enhancement can be attributed to the crucial role of alloying elements in modifying the electrical and geometric characteristics of catalysts. Additionally, M. S. Akkus [37] investigated the influence of thin films of Ni and Ni-Cr alloy, fabricated through the magnetron sputtering process, on H_2_ production from SBH. The incorporation of chromium into the structure was found to enhance both the catalytic performance and reusability of the catalyst. Under identical experimental conditions, the maximum H_2_ production rates for the Ni and NiCr catalyst systems were determined to be 28,362 mL/min g and 30,608 mL/min g, respectively. This enhancement may be owing to the electronic effects of Cr, which modify the charge distribution within the Ni lattice, and its role as a stabilizing agent against nanoparticle aggregation.

This study reports the synthesis of bimetallic Ni-Cr alloy nanoparticles decorated on CNFs using the electrospinning technique for the dehydrogenation of SBH. The synthesized NFs demonstrated significant catalytic efficacy in facilitating H_2_ release from SBH as well as outstanding stability.

## 2. Experimental Section

### 2.1. Preparation of Bimetallic NFs

A 10 wt% solution of polyvinyl alcohol (PVA; MW = 65,000 g/mol, Sigma Aldrich, Saint Louis, MO, USA) was prepared by dissolving PVA powder in deionized water (DI) at a temperature of 50 °C while stirring vigorously. Subsequently, a stock solution consisting of nickel acetate tetrahydrate (NiAc; Sigma Aldrich, USA) and PVA was created in a weight ratio of 1:3. A specific volume of previously prepared chromium acetate dimer (CrAc; Sigma Aldrich, USA) aqueous solution was added to 20 mL of the NiAc/PVA aqueous solution to obtain final solutions with 5 (NiCr-5), 10 (NiCr-10), 15 (NiCr-15), 20 (NiCr-20), and 25 (NiCr-25) wt% CrAc. The final solutions were stirred for 5 hours at 60 °C, then cooled to room temperature to obtain a clear solution. The solution was then subjected to an electrospinning apparatus, utilizing an 18 kV DC power supply, with a tip-to-collector distance of 17 cm. The resulting NFs were collected from an aluminum foil–encased steel rotating cylinder. The electrospun NF mats were dried under vacuum conditions at a temperature of 50 °C for 24 h. Finally, the mats underwent sintering in an argon atmosphere for 5 hours at a temperature of 850 °C, with a heating rate of 3 °C/min.

### 2.2. Characterization of Synthesized Bimetallic NFs

The NFs produced underwent characterization using established methodologies, which included the use of a JEOL JSM-5900 scanning electron microscope (JEOL Ltd., Tokyo, Japan) and a field-emission scanning electron microscope (FESEM, Hitachi S-7400, Tokyo, Japan) to examine the structure of the synthesized NFs. The chemical structure and crystallinity of the synthesized catalysts were analyzed using X-ray diffraction (XRD) with Cu Kα (λ = 1.54056 Å) radiation, provided by Rigaku Co., Tokyo, Japan. A JEOL JEM-2200FS transmission electron microscope (TEM), operating at 200 kV and equipped with EDX (JEOL Ltd., Japan), was utilized to acquire high-resolution images [38]. An X-ray photoelectron spectroscopy (XPS; AXIS-NOVA, Kratos Analytical, Manchester, UK) examination was conducted under following conditions: the base pressure set at 6.5 × 10^−9^ Torr, resolution (pass energy) set at 20 eV, and scan step set at 0.05 eV/step.

### 2.3. Dehydrogenation of SBH

The catalytic activity of the synthesized bimetallic nanofibers in the hydrolysis of SBH (NaBH_4_; Sigma Aldrich, USA) was evaluated using a water-filled gas burette system. The apparatus configuration has been described in other sources [39]. A three-neck flask was utilized, containing an aqueous alkaline solution of SBH. The solution within the flask was agitated using a Teflon-coated stirring bar and included a specific number of NFs. The produced H_2_ was assessed using the water displacement method. The generation rate of H_2_ was investigated for various catalytic NFs and SBH concentrations to determine the reaction rate. Additionally, the activation energy associated with the catalytic process was determined by assessing the catalytic activity at varying reaction temperatures. The flask was placed in a vessel containing silicon oil equipped with a thermostat to facilitate temperature adjustments. The long-term durability of bimetallic NFs in the hydrolysis of SBH at room temperature was also investigated. Furthermore, a controlled test was conducted under identical reaction conditions, excluding any catalytic NFs, to assess the self-hydrolysis of SBH.

## 3. Results and Discussion

### 3.1. Physicochemical Characterization of Bimetallic NiCr@CNFs

Researchers commonly utilize electrospinning technology for its cost-effectiveness, efficiency, and capacity to fabricate outstanding nanofibrous structures from a range of metals, bimetals, trimetals, and their oxides [40]. The electrospinning technique is notable for its ability to produce continuous nanofibrous structures that exhibit high porosity and surface area, which enhance mass transfer and reaction kinetics, making electrospun materials highly effective in catalysis. The versatility of the technique facilitates the synthesis of diverse metals, bimetals, and their oxides into fibrous structures. The choice of metal precursors is crucial as they must withstand polycondensation, an essential step for creating an electrospinnable sol-gel with the appropriate polymer composition. Metal alkoxides typically consist of precursors that undergo hydrolysis and polycondensation to form a cohesive network. Metal salts, such as chlorides, nitrates, and acetates, can undergo hydrolysis and polycondensation, resulting in the formation of gel networks. Acetate has demonstrated its efficacy as a superior salt in this process. Yousef et al. [41] delineate the polycondensation reaction as follows:(1)
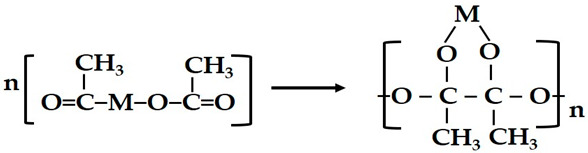


In this reaction, the atom designated as M represents a metal. Consequently, the metal precursors serve to produce highly structured electrospun NFs.

The dissolution of the anions of metal acetate at high temperatures under vacuum and in the presence of argon gas results in the formation of reducing gases (e.g., Co and H_2_). These gases aid in the reduction of acetate and metal oxides, leading to the formation of pure nickel and chromium and/or their alloys. The generation of nickel and chromium can be articulated through the following reactions [42]:

(2) $$Ni(CH_{3}COO{)}_{2}.4H_{2}O\;\;\;\;\;\;\;\;\;\;\;\;\;\;\;\;\;\;\;\;\;\;\;\;\;\to 0.86Ni(CH_{3}COO{)}_{2}.0.14Ni(OH{)}_{2}+3.72H_{2}O\;\;\;\;\;\;\;\;\;\;\;\;\;+0.28CH_{3}COOH$$

(3) $$0.86Ni(CH_{3}COO{)}_{2}.0.14Ni(OH{)}_{2}\to NiO+NiCO_{3}+H_{2}O+CH_{3}COCH_{3}$$

(4) $$NiCO_{3}\to NiO+{CO}_{2}$$

(5) $$NiO+CO\to Ni+{CO}_{2}$$

(6) $$Cr(CH_{3}COO{)}_{2}.2H_{2}O\to 2Cr\left(OH\right)\left(CH_{3}COO\right)+2CH_{3}COOH$$

(7) $$Cr\left(OH\right)\left(CH_{3}COO\right)\to 0.5CrO+0.5Cr{CO}_{3}+0.5H_{2}O+0.5CH_{3}COCH_{3}$$

(8) $$Cr{CO}_{3}\to CrO+{CO}_{2}$$

(9) $$CrO+CO\to Cr+{CO}_{2}$$

**Figure 1:**
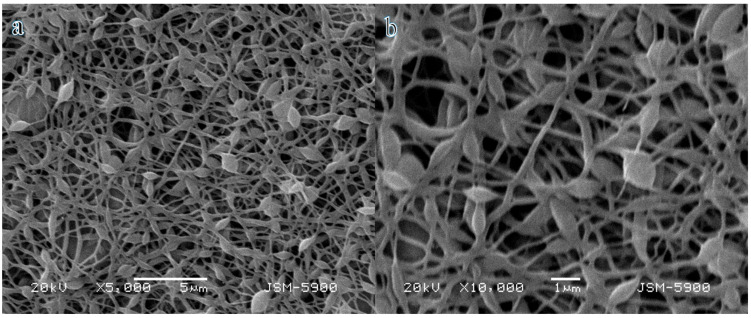
(**a**) Low- and (**b**) high- magnification SEM images of bimetallic NiCr@CNFs.

Various metal acetates were utilized to synthesize inorganic NFs exhibiting excellent morphology. The calcined NFs demonstrated commendable morphology of electrospun NF mats, as illustrated in the SEM images (Figure 1a,b). These images reveal NFs with a pronounced bead-like morphology and a well-defined nanofibrous architecture. Furthermore, a rough texture was observed as the nanoparticles grew and intertwined, potentially forming a network of nanopores. Notably, the nanofibrous morphology exhibited no alterations after the calcination process, as illustrated in the figures. Despite the calcination process occurring in an argon atmosphere, the calcined electrospun nanofibers maintained their structural integrity.

**Figure 2:**
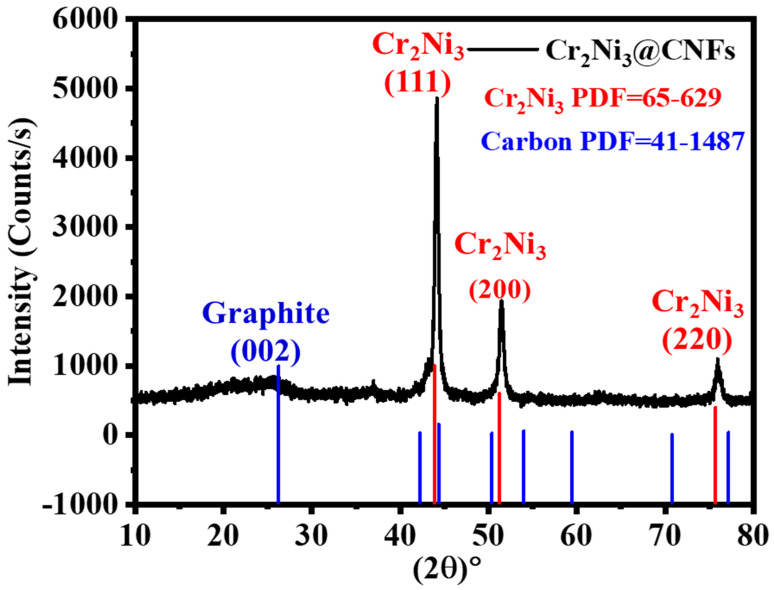
XRD of obtained powder after calcination of electrospun NF mats in argon atmosphere at 850 °C.

XRD studies were conducted on the synthesized NFs to evaluate their bonding characteristics and phase formations. Figure 2 depicts the XRD patterns corresponding to the NiCr@CNFs. Nickel and chromium are neighboring elements within the same period of the periodic table. Thus, there is a solid solution with a concentration of up to 30 wt% at ambient temperature. Beyond this threshold, a eutectic solution is formed. Due to the high melting points of nickel and chromium, which are 1455 °C and 1907 °C, respectively, neither metal will evaporate during the carbonization process. Furthermore, the prepared solution utilized a significant quantity of Ni and Cr precursors. The binary NiCr phase diagrams indicate that chromium has considerable solid solubility in cobalt and nickel. Consequently, the identified peaks in the XRD data demonstrate overlapping characteristics. Moreover, the NiCr alloys exhibit unique crystal structures when contrasted with pure metals. The analysis of the XRD pattern enables the identification of Ni, revealing peaks at 2θ values of 44.5°, 51.8°, and 76.4°, corresponding to crystal planes (111), (200), and (220), respectively (JCDPS #04-0850). The identification of Cr was achieved through the XRD pattern analysis, with peaks observed at 2θ values of 44.4°, 64.6°, and 81.72°. These peaks match the crystal planes (110), (200), and (211), respectively (JCDPS #06-0694). The XRD results of the formed NFs indicate overlapping reflection peaks of Ni and Cr, accompanied by slight variations in their angular positions. The incorporation of the relatively larger Cr atoms led to an increase in the d-spacing within the Ni lattice. The observed 2θ values of 43.9°, 51.2°, and 75.7° exhibit a strong correlation with the crystallographic planes (111), (200), and (220) of Cr_2_Ni_3_, respectively (JCPDS #65-629). The peaks observed in the XRD pattern (Figure 1) can be attributed to the presence of free Ni, Cr, and the Cr_2_Ni_3_ alloy. The binary NiCr phase diagrams demonstrate a significant Cr solid solubility in Ni [43]. Furthermore, a notable broad peak is observed at an angle of 2θ~26°, which signifies an experimental d-spacing of 3.37, substantiating the presence of carbon by this peak (d (002), PDF#; 41-1487) [44]. The results demonstrate the development of bimetallic NiCr nanoparticles on CNFs.

**Figure 3:**
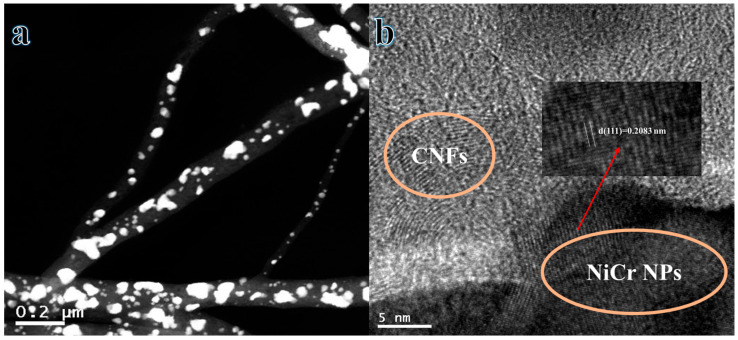
(**a**) TEM (**b**) and HR TEM images of calcined electrospun NF mats.

Figure 3a presents a detailed STEM image of the nanofibers. Numerous light spots from nanoparticles are inspired and distributed uniformly on the rough surface of the NFs. Additionally, HRTEM images serve to ascertain the crystallinity of the synthesized NFs (Figure 3b). The distinct visibility of the spacing between the parallel crystal planes in Figure 3b confirms that the nanoparticles and NFs show exhibit a crystalline structure. The distance between the parallel crystal planes is 0.2083 nm for (111), which is predominantly consistent in the nanoparticles depicted in Figure 3b, thereby corroborating the existence of a singular phase as indicated in the XRD analysis. Consequently, the crystalline nanoparticles and NFs matrix can be attributed to the identified Cr_2_Ni_3_NPs and graphite, respectively.

**Figure 4:**
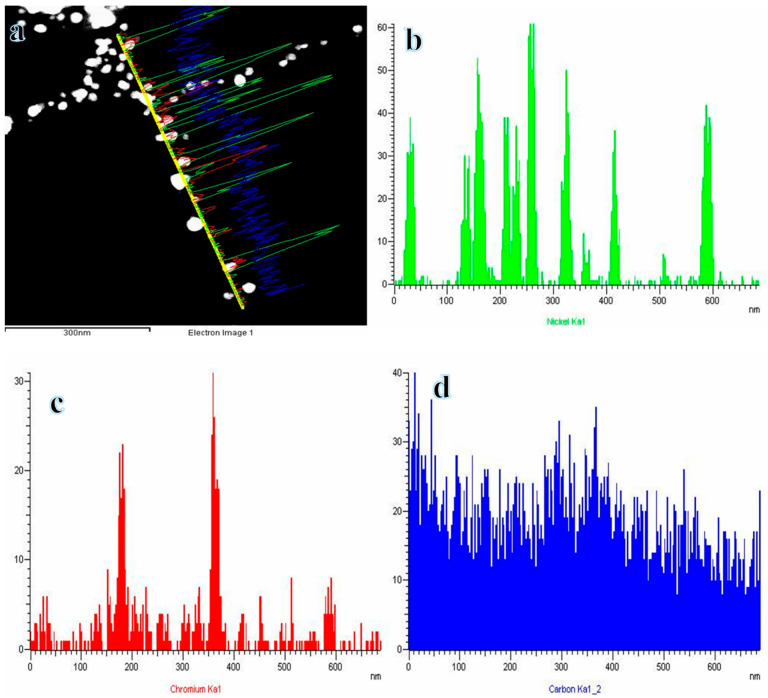
(**a**) STEM image and the corresponding line TEM EDX analysis for (**b**) Ni, (**c**) Cr, and (**d**) C of calcined electrospun NF mats.

TEM-EDX was conducted to analyze the elements and chemical composition of the synthesized NFs. The STEM results (Figure 4a) indicate that the nanoparticles are encapsulated within the NF matrix. The TEM-EDX analysis corresponds to the line depicted in Figure 4a, as shown in Figure 4b–d, which demonstrate that both Ni and Cr nanoparticles are evenly distributed throughout the selected line within the specified area. Thus, a consistent distribution of Ni and Cr occurs, signifying the formation of an alloy. This finding aligns with the XRD findings, confirming the synthesis of the Cr_2_Ni_3_phase. Consequently, the synthesized nanoparticles and NFs were attributed to the Cr_2_Ni_3_alloy and CNFs, respectively. Furthermore, the Cr_2_Ni_3_NPs were effectively organized within a thin crystalline layer of CNFs. This layer was consistently observed at every location along the selected line. The incorporation of carbon has the potential to enhance catalytic activity, material conductivity, and stability. Additionally, CNFs exhibit significant adsorptive capacity, improving the interaction between the target substance and the catalytic material, while facilitating the efficient release of the product due to their inherent porosity. The adsorption of SBH by CNFs is a straightforward process that enhances electron transfer, thereby significantly aiding in the separation of hydrogen atoms.

**Figure 5:**
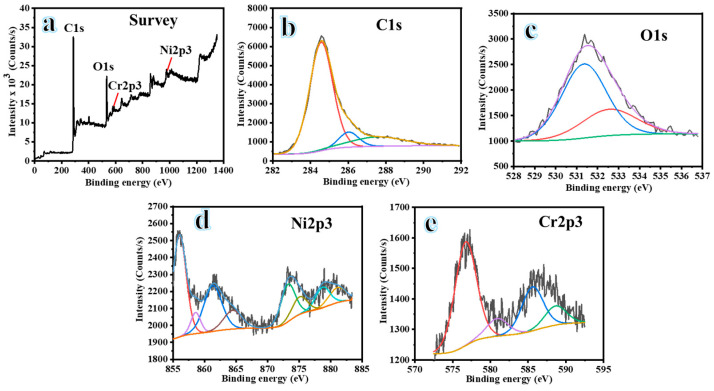
XPS spectra analysis; survey spectrum of (**a**) Cr_2_Ni_3_@ CNFs; (**b**) C1s; (**c**) O1s; (**d**) Ni2p; and (**e**) Cr2p.

XPS primarily identifies the chemical states of material surfaces up to a thickness of 10 nm. We observed significant peaks of C1s, O1s, Cr2p, and Ni2p in the full scan XPS spectra. Figure 5b,c illustrate the detection of C1s and O1s at 284.56 eV and 531.50 eV, respectively. The electron-binding energies of Ni 2p3/2 and Ni 2p1/2 were detected at 856.05 eV and 873.56 eV, respectively (Figure 5d); these peaks are indicative of the oxide form of Ni. The shake-up satellite peaks at 861.66 eV and 879.39 eV in the spectra are associated with Ni^2+^, suggesting the presence of NiO. Figure 5e displays the XPS spectrum of Cr, with two peaks at 576.78 eV and 586.35 eV, attributed to Cr 2p3/2 and Cr 2p1/2, respectively, indicating that Cr exists in oxidized forms [26]. The findings demonstrate the oxidation of the Ni and Cr surfaces of the Cr_2_Ni_3_ alloy to NiO and Cr_2_O_3_, which is attributed to the exposure of the surface sample to air during preparation. The formation of a thin oxide layer is inevitable due to the sample’s exposure to air during preparation. S. K. Singh et al. [45] eliminated the oxygen layer generated via argon sputtering prior to XPS analysis. Consequently, the bulk alloy phase remains preserved, as confirmed by XRD conducted at a thickness of several micrometers. It is important to note that the surface oxidation layer may affect the material’s catalytic activity by providing active sites for various reactions.

### 3.2. Catalytic Activity Studies of Prepared Catalysts

#### 3.2.1. H_2_ Evolution Efficacy of Bimetallic NiCr Alloy Nanoparticles-Decorated CNFs Catalysts in the Dehydrogenation of SBH

**Figure 6:**
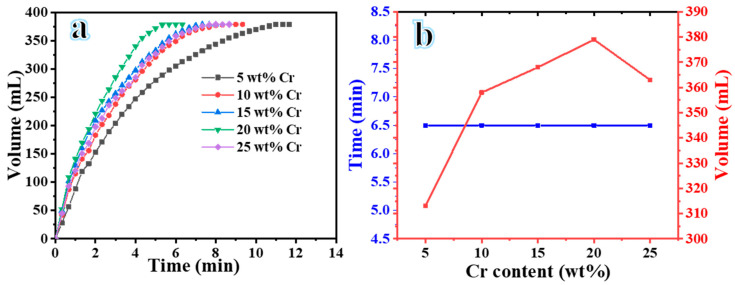
(**a**) The influence of various NiCr@CNFs catalysts on H_2_ production and (**b**) the influence of Cr % on H_2_ production.

This study represents a pioneering effort to examine the impact of bimetallic NiCr alloy@CNFs on the H_2_ evolution of an aqueous solution of SBH. A sample weighing 100 mg was evaluated for its H_2_ evolution efficacy at 25 °C and 1000 rpm using an aqueous solution of 1.5 wt% SBH. The results depicted in Figure 6a show the exceptional catalytic activity of the NiCr-20 catalyst, facilitating the completion of the reaction in just 6.5 min. The results indicate that H_2_ evolution is significantly more rapid with the NiCr-20 catalyst compared to the other formulations. Li et al. [46] discovered that introducing a suitable proportion of Cr to the Co-Cr-B/nitrogen-doped graphene catalysts resulted in a shift in the position of the Fermi level, which subsequently altered the free energy and the position of the valence band center. This modification enhanced the efficiency of H_2_ production from the dehydrogenation of SBH. Figure 6b demonstrates the effect of Cr content on H_2_ production, with the NiCr-20 catalyst exhibiting the highest volume of H_2_ within the same timeframe. The distinctive increase in H_2_ generation rate could be attributed to the synergistic effect of Ni and Cr in the prepared NFs. M. S. Akkus [37] found that incorporating Cr into the Ni structure enhances both the catalytic activity and reusability of the fabricated catalyst in H_2_ generation from SBH. Moreover, the CNFs contribute to this remarkable performance. The CNFs exhibit exceptional absorptive capacity and surface area, facilitating interaction with a widely dispersed active catalyst, which leads to the generation of numerous active sites [47]. The electrospun CNFs are recognized for their highly porous structure, promoting efficient interaction between reactants and active metals, thereby facilitating H_2_ evolution [42]. The nano-fibrous structure consequently led to a notable enhancement in surface area and active sites for SBH dehydrogenation [48].

#### 3.2.2. Influence of Bimetallic NiCr Alloy Dosage on H_2_ Evolution

**Figure 7:**
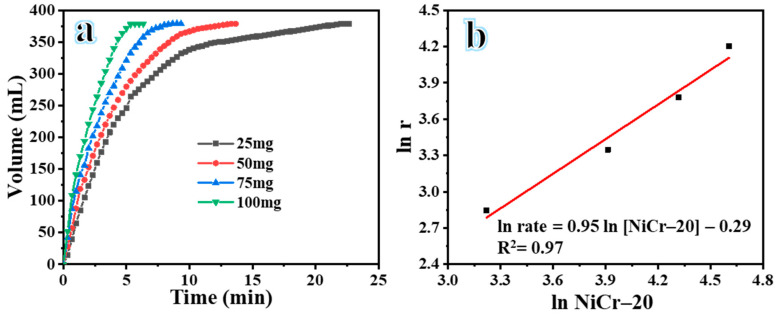
(**a**) The influence of various NiCr-20@CNF dosages on the H_2_ production and (**b**) logarithmic correlation between the rate of H_2_ evolution and NiCr-20@CNFs.

The NiCr-20 catalyst was employed to investigate the influence of catalyst dosage on H_2_ evolution. At a temperature of 25 °C and 1000 rpm, four distinct dosages of NiCr-20@CNFs catalysts (25 mg, 50 mg, 75 mg, and 100 mg) were evaluated, and the H_2_ evolution was observed in the presence of an aqueous solution of 1.5 wt% SBH. Figure 7a illustrates the H_2_ evolution profile of the SBH solution when catalyzed by varying dosages of NiCr-20 catalysts. The figure clearly demonstrates that H_2_ evolution from the SBH solution consistently increases as the catalyst dosage increases. This is attributed to the enhanced accessibility of surface active sites, which can improve the dehydrogenation process. Figure 7b illustrates the logarithmic plot correlation between the rate of H_2_ evolution from the aqueous solution of SBH and the dosage of catalyst utilized. The figure indicates a direct correlation between the dosage of NiCr-20@CNF catalyst and the rate of H_2_ evolution from the aqueous solution of SBH. The estimated slope of the line was found to be 0.95. These findings suggest that SBH hydrolysis utilizing a NiCr-20@CNFs catalyst complies with first-order kinetics.

#### 3.2.3. Influence of SBH on the Efficacy of H_2_ Evolution

**Figure 8:**
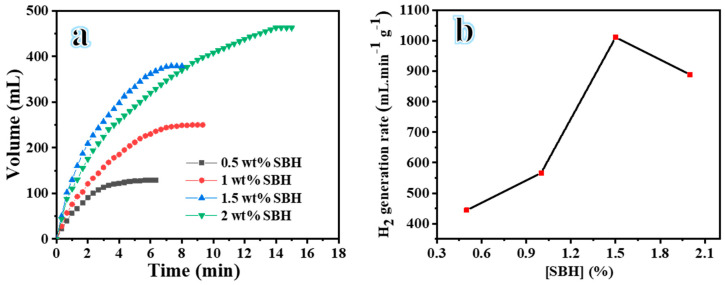
(**a**) H_2_ evolution using different concentration of SBH and (**b**) H_2_ generation rate versus concentrations of SBH.

The figure shows the impact of varying SBH (0.5 wt%, 1.0 wt%, 1.5 wt%, and 2.0 wt%) on H_2_ evolution, utilizing 100 mg of NiCr-20@CNFs, at 25 °C and 1000 rpm. Figure 8a,b illustrate that the rate of H_2_ evolution remains relatively stable as the concentration of SBH increases from 0.5 wt% to 1.0 wt%. However, there is a notable increase in the reaction rate when the SBH concentration rises from 1.0 wt% to 1.5 wt%. This phenomenon may be attributed to the elevated levels of BH4^−^, which aid in the release of H_2_. Additionally, an increase in the H_2_ evolution rate is noted when transitioning from 1.5 wt% to 2.0 wt%. This increase in SBH concentration leads to a corresponding increase in H_2_O consumption, resulting in a significant generation of NaBO_2_. This process contributes to the viscosity of the solution, subsequently reducing the contact area between the BH4^−^ and the NiCr-20@CNFs catalyst, ultimately leading to a diminished H_2_ evolution rate [49].

#### 3.2.4. Influence of Temperature on H_2_ Evolution

**Figure 9:**
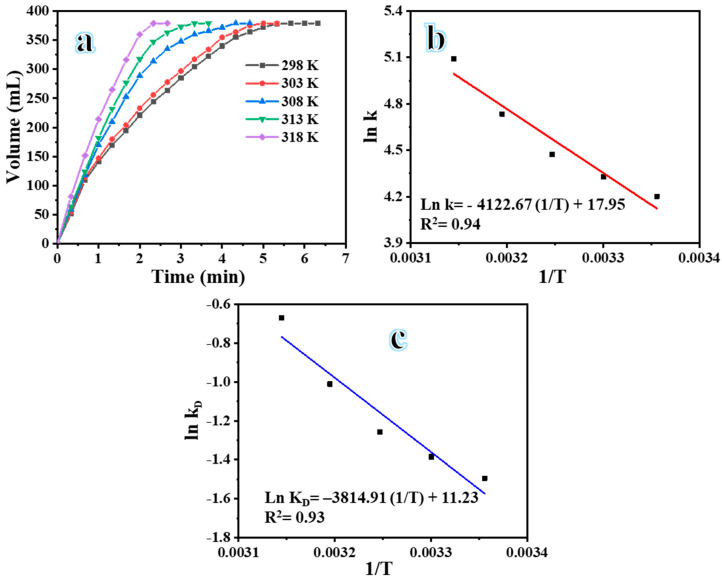
(**a**) The influence of temperature on H_2_ evolution; (**b**) correlation of logarithmic K and 1/T; and (**c**) correlation of logarithmic K_D_ and 1/T.

The H_2_ evolution of the aqueous solution of 1.5 wt% SBH was determined at five distinct temperatures (298 K, 303 K, 308 K, 313 K, and 318 K) using 100 mg of the NiCr–20@CNFs catalyst. The activation energy (E_a_) of SBH dehydrogenation was determined based on the experimental findings. Figure 9a demonstrates that H_2_ evolution at 298 K requires 6.5 min for completion, whereas at 318 K, the same reaction is completed in just 2.5 min. These results indicate that as the temperature of the SBH solution rises, the rate of H_2_ evolution increases. The rise in temperature leads to a greater contact area between the catalyst and the SBH solution, resulting in a faster dissolution of the by-product NaBO_2_. Moreover, this phenomenon facilitates the rapid evolution of H_2_ from the catalyst surface [50]. Figure 9b,c present Arrhenius and Eyring plots, respectively, which were used to determine the thermodynamic parameters at each temperature. The rate constants were calculated by assessing the slope of the linear portion of each plot in Figure 8. The activation energy for the hydrolysis process was estimated to be 34.27 kJ mol^−1^, with ΔH being 31.71 kJ mol^−1^ and ΔS being 93.28 J mol^−1^ K^−1^. The negative ΔS suggests that the rate-determining step relies on an associative activation mode rather than a dissociative mode. The obtained E_a_ is comparatively lower than that of several catalysts reported in previous studies (Table 1). This lower E_a_ can be attributed to the synergistic interaction between nickel and chromium. The findings indicate that NiCr-20@CNFs exhibits promising catalytic activity for the generation of H_2_ from SBH.

#### 3.2.5. Stability of Bimetallic NiCr Alloy Catalyst

**Figure 10:**
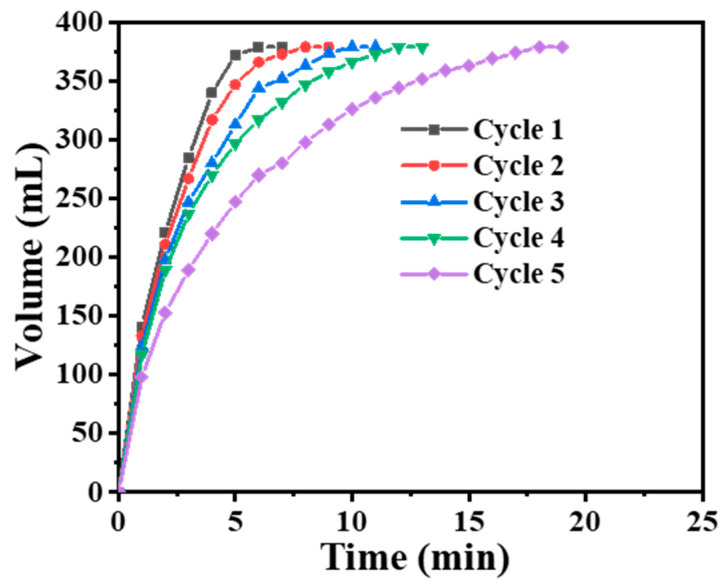
NiCr-20@CNFs reuse performance test.

Figure 10 depicts the five-cycle examination of the 100 mg NiCr-20 catalyst utilized for the dehydrogenation of a solution of 1.5 wt% SBH at 25 °C and 1000 rpm. The figure indicates that the NiCr-20@CNFs catalyst decreases the H_2_ evolution rate to 37.89% of the initial value following five cycles of testing. The rate of H_2_ evolution during the dehydrogenation of SBH exhibits a distinct decline. Thus, the NiCr-20@CNFs catalyst demonstrates diminished cycling stability. This observed phenomenon could be attributed to the accumulation of NaBO_2_ produced after multiple cycles, which obscures the catalyst surface and consequently diminishes its catalytic efficacy [37,60]. The NiCr-20@CNFs catalyst could undergo significant depletion over multiple cycles, leading to an inadequate amount available for reaction with the SBH solution, ultimately resulting in a decreased H_2_ evolution rate.

## 4. Conclusions

The catalytic NFs were successfully synthesized using a straightforward electrospinning technique. Characterization confirmed the formation of NiCr alloy nanoparticles on CNFs. By adjusting the amount of Cr doped into the Ni structure, we were able to enhance catalytic activity, alter electronic properties, and significantly improve hydrogen evolution performance. Notably, the NiCr-20 sample outperformed the other formulations in catalytic efficiency. Kinetic studies revealed that hydrogen production followed a first-order reaction concerning the catalyst quantity, and increasing the concentration of SBH further boosted hydrogen generation. Additionally, higher temperatures led to shorter reaction times. The Ea, ΔS, and ΔH were calculated to be 34.27 kJ mol^−1^, 93.28 J mol K^−1^, and 31.71 kJ mol^−1^, respectively. The catalyst demonstrated good stability, maintaining performance over five cycles. The enhanced catalytic behavior is attributed to the synergistic effect between Ni and Cr, making this approach a promising avenue for the development of advanced Ni-based catalysts.

## Figures and Tables

**Table 1:** The activation energy of Ni-based catalysts for H_2_ production from SBH.

Catalyst	E_a_ (KJ/mol)	Ref.
Ni	42.28	[21]
Ni	71	[51]
Raney Ni	63	[51]
Pd-Ni-B	31.1	[31]
Ni-Co	38	[52]
Ni-Co-B	62	[53]
Co-Ni@ reduced graphene	55.2	[54]
Co-B/Ni	94.5	[55]
Co-Ni-B	33.1	[56]
Co-Ni/AC	68.9	[57]
Co-Ni/MWAC	40.7	[58]
Sm- Ni-Co-P/g-Al_2_O_3_	52.1	[59]
NiCr@CNFs	34.27	This study

## Data Availability

The original contributions presented in the study are included in the article, further inquiries can be directed to the corresponding author.
